# Results of Large-Scale Propagation Models in Campus Corridor at 3.7 and 28 GHz

**DOI:** 10.3390/s21227747

**Published:** 2021-11-21

**Authors:** Md Abdus Samad, Feyisa Debo Diba, Young-Jin Kim, Dong-You Choi

**Affiliations:** 1 Department of Information and Communication Engineering, Chosun University, Gwangju 61452, Korea; masamad@chosun.kr (M.A.S.); feyisa2006@yahoo.com (F.D.D.); 2 Department of Electronics and Telecommunication Engineering, International Islamic University Chittagong, Chittagong 4318, Bangladesh; 3 Department of Electronics and Communication Engineering, Adama Science and Technology University, Adama 1888, Ethiopia; 4 Department of e-Sports, Chosun College of Science & Technology, Gwangju 61453, Korea; yjkim@cst.ac.kr

**Keywords:** wave propagation, indoor corridor, long corridor, CI model, CIF model, FI model, ABG model

## Abstract

The indoor application of wave propagation in the 5G network is essential to fulfill the increasing demands of network access in an indoor environment. This study investigated the wave propagation properties of line-of-sight (LOS) links at two long corridors of Chosun University (CU). We chose wave propagation measurements at 3.7 and 28 GHz, since 3.7 GHz is the closest to the roll-out frequency band of 3.5 GHz in South Korea and 28 GHz is next allocated frequency band for Korean telcos. In addition, 28 GHz is the promising millimeter band adopted by the Federal Communications Commission (FCC) for the 5G network. Thus, the 5G network can use 3.7 and 28 GHz frequencies to achieve the spectrum required for its roll-out frequency band. The results observed were applied to simulate the path loss of the LOS links at extended indoor corridor environments. The minimum mean square error (MMSE) approach was used to evaluate the distance and frequency-dependent optimized coefficients of the close-in (CI) model with a frequency-weighted path loss exponent (CIF), floating-intercept (FI), and alpha–beta–gamma (ABG) models. The outcome shows that the large-scale FI and CI models fitted the measured results at 3.7 and 28 GHz.

## 1. Introduction

By 2023, there will be over three times as many devices linked to the Internet protocol network than there will be human beings [1]. Humans will use many devices to access multimedia content, services, and data [2] through wireless networks. One of the most effective decisions to facilitate enhanced mobile broadband (eMBB) services to these huge devices is to relocate data transmissions into an under-utilized nontraditional range, where huge bandwidths are available [3]. As such, 5G-new radio provides a high-data-rate service which is achieved by the maximum spectral efficiency [4] and the use of millimeter wave (mmWave) [5]. A large part of all the eMBB services will be for the indoor environment, where people stay for different activities such as studying, working, living, leisure, or healing purposes. With the large bandwidth within the mmWave spectrum, a significant component of the 5G mobile network, the mmWave was proposed to enable multi-gigabit telecommunication, visual services, for example, ultra-high-definition video and high-definition television [6,7,8,9] and multi-gigabit communication, such as device-to-device communication [7,10,11].

However, the mmWave frequency band suffers from high building penetration loss and a more significant delay spreading from outdoor to indoor coverage networks, as reported in [12,13,14,15]. Presently, 5G primary service locations with high-frequency bands are anticipated to be environments for, e.g., internal hot-spots and micro-cells [16]. Consequently, a separate indoor transmission system can promise to ensure better spectral efficiency (and better eMBB services) for indoor network demands. However, only deploying the separate transmitter in indoors settings will not be helpful if the proper transmission of the electromagnetic wave is not modeled according to its internal infrastructure settings and the used materials [17]. Therefore, it needs to find a proper propagation model matching its structure, which can help achieve high-speed data transfer service as per the demands of indoor users.

Indoor mmWave propagation path loss models are critical components to ensure quality network access for indoor environments [18]. Consequently, path loss modeling for indoor users is vital to the network design, planning, performance evaluation, and implementation. Therefore, several organizations [19,20] are now involved in designing mmWave channel models.

The path loss modeling is a mathematical analysis of the radio wave propagation process that considers the signal-to-noise and interference ratios and spectral efficiency to provide the network coverage area [21]. In addition, as the mmWave band is the most promising carrier in wireless propagation channels and has higher wall penetration loss, precise path loss models are required [18]. Therefore, several path loss models based on the statistical or empirical approach, such as the Okumura–Hata model [22] or the Coopération Européenne dans le Domaine de la Recherche Scientifique et Technique (COST) 231 path loss model [23] that consider the number of traversed floors, the number of lightweight interior partitions, windows, and the number of concrete or brick internal walls [23], and other models [24,25], are proposed in the literature. Most of these models consider the attenuation due to individual elements that hinder the radio wave propagation. Nevertheless, recently, large-scale attenuation parameters have been realized for the propagation modeling of the radio wave in indoor environments, rather than considering the attenuation due to individual elements [21,26,27,28].

In the CI, FI, and ABG model, some parameters relate to the path loss as a function of either distance or frequency parameters or parameters of both distance and frequency. In such a model, the path loss and the parameters’ dependency can be realized through coefficients such as “path loss exponent”, “α”, and “β” [29]. In many respects, the indoor environment differs substantially from the outdoor environment [17]. As a result, the interior path loss models must account for changes in floor layout, construction materials, the variety and quantity of office equipment, numbers of persons and their movements, and the density of utilizing the wireless network in the vicinity. Furthermore, multipath propagation and normal fading and path loss due to distance and physical phenomena such as interference, reflection, refraction, dispersion, and penetration can affect the received signal’s characteristics [30].

In the literature, many investigations of radio wave propagation have been studied either through a real measured experiment setup condition [21,26,27,31] or through simulation-based study [32]. However, in both types of analysis, currently, the maximum path length in the corridor environment studied in [33] is approximately 100 m. In [31], a total 77 m path length was studied, but, and 25 m was the NLOS path length in an “L-shaped” corridor. As previously noted, several initiatives have been undertaken in the literature to assess path loss in indoor corridor environments. Among these studies, a more than 30 m corridor length measurement result was reported in [26,27,32,33,34,35,36,37,38,39,40,41,42]. In Table 1, we present all of these research outcomes’ investigated link types, frequency bands, and distances studied.

Most recent research has been concentrated on the 28, 38, and the 81–86 GHz E-band [18]. New York University (NYU) and the mobile and wireless community enablers for the twenty twenty information society (METIS) have reported ongoing campaigns to estimate 5G channels and modeling for the 20–70 GHz range [43,44]. However, several research outcomes are available at 3.7 and 28 GHz [33,35,37,38,39] bands, which is not significant enough, and there is still a research scope to determine the suitable path loss models in different environments at these frequency bands. The above-stated indoor radio wave propagation prediction differs in certain respects from the outdoor one [17]. The extent of the network coverage is determined in the indoor instance by the construction geometry, and the boundaries of the structure itself affect radio wave propagation [45,46]. Frequency reuse between the floors of the same building is also typically wanted, adding a third dimension to interference difficulties. Small changes in the immediate radio path environment may significantly influence propagation features [47]. Consequently, detailed structural variation-based indoor corridor propagation modeling is also necessary.

The 3.5 GHz bands are currently undergoing the roll-out operation with the 5G network in Korea, and 28 GHz bands are probably the future frequency bands to be deployed in Korea [48] and the USA [49]. This study designed an experiment to study the path loss at 3.7 and 28 GHz frequency bands in the long indoor corridors that have not been studied to date. In addition, we make the following unique contributions:

- We measured the wave propagation in a 90–260 m-long corridor in the university campus, and the measured path losses were modeled with the CI, CIF, FI, and ABG methods;
- The parameters of the CI, CIF, FI, and ABG models were calculated using the MMSE-based optimization method;
- The resulted coefficients of CI, CIF, FI, and ABG models were analyzed.

This study is organized as follows: Section 2 provides experimental scenarios and experimental parameters descriptions. Section 3 provides information about the CI, CIF, FI, and ABG models, and Section 4 contains an analysis of large-scale path loss models and the experimental data. Section 5 discusses the results obtained from the experimental results. The conclusions are presented in Section 6.

## 2. Measurement Campaign

This section describes the measuring technique, the channel formation, the measurement environment, the instruments utilized for the measurement campaign, and the signal pre-processing steps.

### 2.1. Measurement Equipment

**Figure 1:**
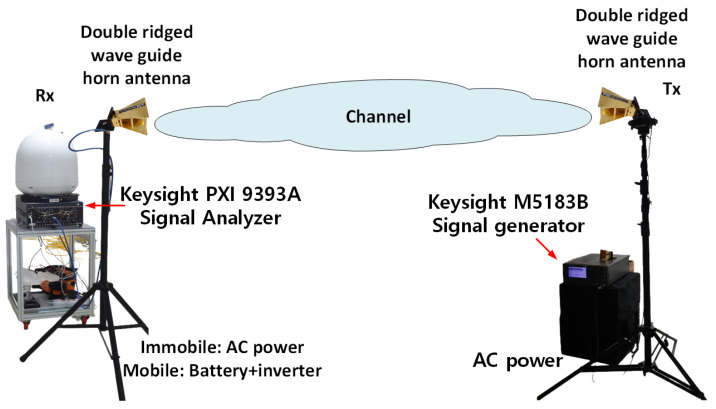
Channel sounder architecture.

This section describes in detail the channel sounder and the scenarios incorporated in the measurement operations. We utilized the M5183B keysight signal generator (at Tx side), keysight PXI 9393A signal analyzer (at Rx side), and two-directional horn antennas. The channel sounder and the other devices utilized in our campaign are shown in Figure 1. A clean and accurate alternative to the analog PSG is the N5183B MXG (PSC and MXG are product series names of Keysight Technologies, Inc., Santa Rosa, CA, USA) microwave analog signal generator with benefits in size and speed. It can precisely yield the required purity of spectra, the desired output power level. In addition, the device is compact with just two racks of units and can nonetheless maintain rigorous performance with near-PSG performance levels. The module can also be used to test radar modules and methods giving the best-in-class state noise of ≤−124 dBc/Hz (10 kHz offset) with −75 dBc spurious (at 10 GHz). The use of the MXG can further expedite the calibration process with a best-in-class switching rate of approximately 600 μs.

In the measurement campaign, the keysight’s signal generator MXG N5183B was used as a transmitting source. In the receiver end, the keysight’s signal analyzer PXI 9393A was used to receive and process the received signal, operating in the frequency range of 9 kHz–50 GHz. Horn antennas with gains of 10, 20, and 20 dBi were employed in the experimental setup for the 3.5 GHz directional antenna, 28 GHz directional antenna, and the 28 GHz TAS antenna, respectively. H–H co-polarization was used for the horn antennas and throughout all measuring experiments. Figure 1 provides the measurement system with the elements, and Table 2 shows the additional operational parameters of the system.

#### 2.1.1. Signal Generators

The transmitter (MXG N5183B) can generate continuous sinusoidal wave (CW) analog signals as well as a wide range of signals from 9 kHz to 40 GHz [50]. The frequency switching can be implemented using a “listing mode” type operation where the switching time is 600 μs. The sweep mode is also a “listing type” as a frequency switching technique, and it changes stepwise. It can generate the minimum power −130 dBm, and the maximum power can be +20 dBm (say at 1 GHz). The signal generator has a level accuracy of approximately ±0.7 dB. In the SSB mode operation, the phase noise can be at 1 GHz with a 20 kHz offset setting is −124 dBc/Hz. It can generate harmonics at 1 GHz up to −55 dBc, and non-harmonics (at 1 GHz) up to −100 dBc. Furthermore, it can generate ten *n*s pulse width and pulse modulation phase deviation (maximum in standard mode) in the range of 0.5–64 rad.

#### 2.1.2. Signal Analyzer Properties

The vector signal analyzer’s core comprises the following components: M9308A PXIe synthesizer, M9365A PXIe down-converter, and M9214A PXIe intermediate frequency (IF) digitization. The signal analyzer we used is the PXI 9393A [51]. This device can be used to analyze the frequency range from 9 kHz to 8.4, 14, 18, or 27 GHz and in an extended mode in the range of 3.6–50 GHz. This can analyze the signal with 40, 100, or 160 MHz. The absolute amplitude accuracy is ±0.13 dB, and the frequency switching is approximately smaller than 135 µs. It can display the average noise level up to −168 dBm/Hz. The third-order inter-modulation is approximately +31 dBm.

### 2.2. Environmental Scenario Descriptions of the Measurement Campaigns

#### 2.2.1. Corridor Wall and Floor Materials

The measurement operations were conducted inside the CU, in the 10th floor straight corridor of the IT convergence building and the straight corridor of the main building. The indoor ambiance restrained the signaling system and directed it in different directions that affect the electrical phenomena of the received signal. Several small fire extinguishers, a hot and cold drinking water supply system, and an automatic drinking beverage dispenser made of metal were located in the extended portion of the corridor in the middle of the IT convergence building. The hallway is comprised of brick walls, a square-tiled floor, metal doors, and metal grill structures to hold the glass at the two long corridor ends. The sidewalls of the IT convergence corridor were constructed of lightweight concrete and a false gypsum ceiling. Additional information about the construction materials is given in Table 3.

#### 2.2.2. Corridor Shape Irregularities

**Figure 2:**
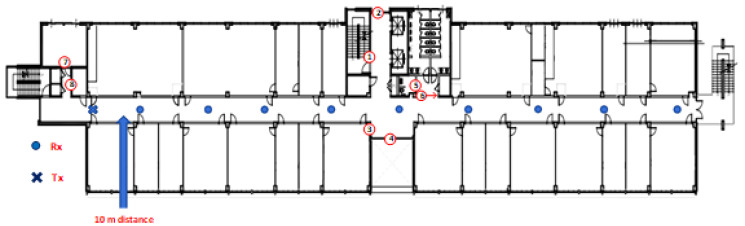
Measurement locations of the transmitter and receiver during the campaign. There are some structural changes in the corridor. These 2-dimensional changes of such spaces are marked with circled numbers and the length of the irregularities are: ➀ 8.894 m; ➁ 2.950 m; ➂ 1.540 m; ➃ 6.55 m; ➄ 2.322 m; ➅ 3.162 m; ➆ 1.371 m; and ➇ 3.267 m.

**Figure 3:**
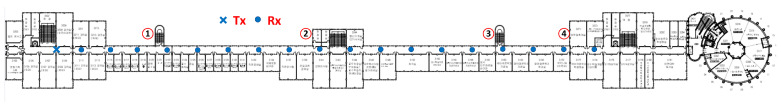
The experimental outlet is on the 3rd floor of the main building corridor. Structures that create irregularities in the corridor are marked with circled numbers: ➀; ➁; ➂; and ➃.

There are four irregular spaces in the IT corridor. Circled numbers in Figure 2 mark all these spaces. In the middle of the IT corridor, there are spaces on both sides of the corridor. On one side, there is a space to accommodate a “beverage vending machine” and in another corner, there is a drinking water purifying system (see around ➂ of Figure 2). On the other side, there are spaces for two elevators on one side, and on the other side, there are emergency stairs (see around ➁ of Figure 2). Another irregularity of the corridor is the restroom spaces marked by the place around ➅ of Figure 2. The fourth irregularity was the place market by the space around ➇ of Figure 2, which was located behind the transmitter during the measurement operation. Furthermore, in the main building corridor, one side was mainly open, and another side was the classroom, research lab, and office, as shown in Figure 3.

#### 2.2.3. Measurement Caution

During the measurement campaign, all the doors and windows were closed. Additionally, no humans were allowed to stay between the transmitter and receiver during the measurement operation. There were no other objects in both corridors except small dustbins made of plastic materials, and there few other things exist that were already reported in Section 2.2.1 (corridor wall and floor materials). The electric lights were switched off in the measuring area to eliminate any possible light impacts on the propagating electromagnetic wave.

#### 2.2.4. Campaigns’ Description

**Figure 4:**
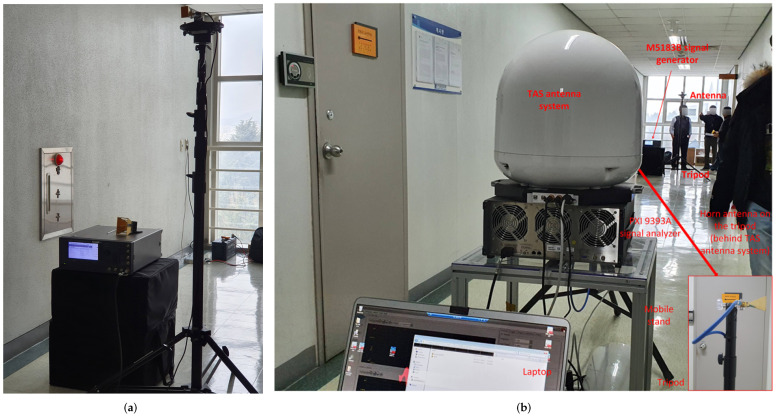
(**a**) The figure shows the location of the transmitter of the measurement campaign in the corridor of the IT convergence building on the 10th floor. The 1.75 m height transmitter was installed at 4.3 m along the wall; (**b**) the figure shows a measurement location while moving the receiver in a particular position along the corridor of the IT convergence building on the 10th floor. The picture includes the Rx horn antenna and the TAS antenna.

The first measurement operations were conducted in the corridor of the IT convergence building (Figure 4a,b). The transmitting antenna (Tx) was installed 5 m from the back wall (along the longitudinal direction) and in the center of the hallway, using a guided horn antenna. We changed the antenna for different frequency experiments where the antenna gains were 10, 20, and 20 dBi for the 3.7 GHz directional antenna, 28 GHz directional antenna, 28 GHz TAS antenna, respectively, as mentioned earlier. The trial data were collected at every 10 m distances of the IT convergence corridor (Figure 2), and 14 m, 20 m, 30 m, 40 m, 50 m, 60 m, 70 m, 80 m, 90 m, 100 m, 120 m, 140 m, 160 m, 180 m, 200 m, 220 m, 240 m, and 260 m of the main building corridor (Figure 3). Following the procedure, we collected nine LOS experimental results at every 3.7, 28, and 28 GHz (TAS) frequencies in the IT convergence corridor and 18 measurement results at every 3.7 and 28 GHz frequencies in the main building corridor.

**Figure 5:**
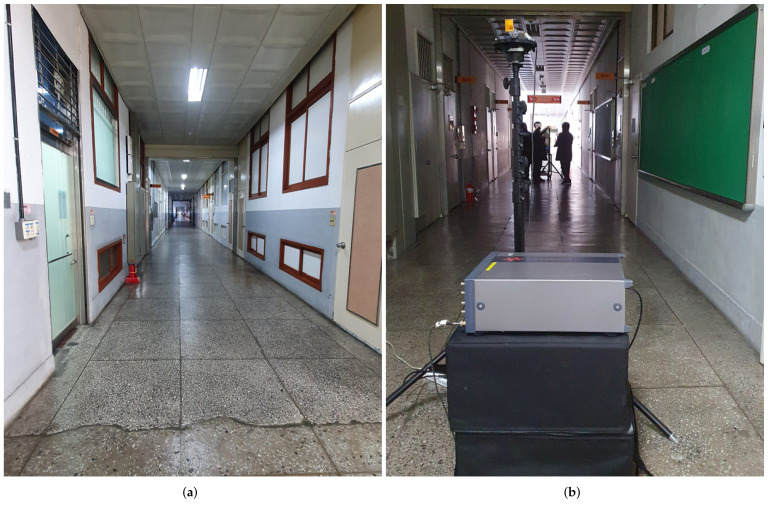
(**a**) The figure shows the 375 m-long corridor without human movement on the 3rd floor of the main building just before the measurement campaign; and (**b**) it shows a measurement scenario of the 3rd floor corridor of the main building.

The second campaign was operated in the main building of the CU (Figure 5a,b), which was recorded as the most extended building in the “Guinness book of records” some years ago [52]. The same procedure of IT corridor measurement was followed to assess the path loss in the corridor as in the IT convergence building, except the TAS receiver data were not measured. The layout of the main building corridor is given in Figure 3.

### 2.3. Data Pre-Processing

Path loss is essential for the development of link budgeting and wireless link coverage in radio channel models. If we denote the transmitted signal power by $P_{Tx}$ and the received signal power by $P_{Rx}$, the path loss of a radio link in dB-scale at each measured data location can be calculated as

(1) $$PL=\left(P_{Tx}+G_{Tx}+G_{Rx}\right)-\left(P_{Rx}+C_{Tx}+C_{Rx}\right)$$

where $G_{Tx}$ and $G_{Rx}$ are the gains of the used antennas and $C_{Tx}$, $C_{Rx}$ are the cable loss at the Tx and Rx sides, respectively.

## 3. Path Loss Prediction Models

In the next section, we discuss the procedure to determine the coefficients of the FI, CI, CIF, and ABG models.

### 3.1. Single-Frequency Propagation

#### 3.1.1. CI Model

The CI model of wave propagation is given by the equation:

(2) $$\begin{array}{cc} {\mathrm{PL}}^{\mathrm{CI}}\left(f,l\right)= & \mathrm{FSPL}\left(f,1m\right)+10n\log_{10}\left(l\right)+X_{\sigma }^{\mathrm{CI}}\left[\mathrm{dB}\right];\;\mathrm{for}\;l\geq 1m \end{array}$$

where $X_{\sigma }^{\mathrm{CI}}\left(\mu ,\sigma^{CI}\right)$ is a Gaussian random variable which is characterized by the standard deviation $\sigma^{CI}$ measured in dB and the mean value of the random variable is zero (μ=0). The free space path loss (FSPL) (f,1m)=$10\log_{10}{\left(\frac{4\pi f}{c}\right)}^{2}$ is the free space path loss with a reference distance of 1 m and *n* is the path loss exponent (PLE). The CI method presents the large-scale channel fluctuations owing to the shadowing effect [29]. The PLE *n* path loss pattern is calculated by the MMSE-based optimization method which matches the data determined to the minimum error (by lowering $\sigma^{CI}$) with the actual physical anchor point, representing the freely available space power transmitted by the *Tx* antenna at the proximity [53]. To determine the optimum PLE, using the MMSE-based optimization technique, Equation (2) can be arranged as

(3) $$\begin{array}{cc} X_{\sigma }^{\mathrm{CI}}= & {\mathrm{PL}}^{\mathrm{CI}}\left(f,l\right)\left[\mathrm{dB}\right]-\mathrm{FSPL}\left(f,1m\right)-10n\log_{10}\left(l\right);\;\mathrm{for}\;l\geq 1m \end{array}$$

assuming that $F={\mathrm{PL}}^{\mathrm{CI}}\left(f,d\right)\left[\mathrm{dB}\right]-\mathrm{FSPL}\left(f,1m\right)$, and $L=10\log_{10}\left(d\right)$, Equation (2) becomes:

(4) $$X_{\sigma }^{\mathrm{CI}}=F-nL$$

the standard deviation of the shadowing factor (SF) is calculated using the MMSE method as follows:

(5) $$\sigma^{CI}=\sqrt{\frac{\Sigma {\left(X_{\sigma }^{CI}\right)}^{2}}{D}}=\sqrt{\frac{\sum {\left(F-nL\right)}^{2}}{D}}$$

where *D* is the number of the Tx–Rx separation distances or the number of recorded different measurement data. To minimize the SF with the standard deviation $\sigma^{CI}$ is commensurate to reducing the term $\sum {\left(F-nL\right)}^{2}$. If $\sum {\left(F-nL\right)}^{2}$ is lessened, the derivative about *n* should be zero:

(6) $$\begin{array}{c} d\sum {\left(F-nL\right)}^{2}\;/\;dn=\sum 2L\left(nL-F\right)=2\sum L\left(nL-F\right)=2\left(n\sum L^{2}-\sum LF\right)=0 \end{array}$$

Therefore, from Equation (6):

(7) $$n=\frac{\sum FL}{\sum L^{2}}$$

Thus, the smallest SF standard variation for the CI model is:

(8) $$\sigma_{min}^{CI}=\sqrt{\frac{\sum {\left(F-nL\right)}^{2}}{D}}$$

The calculated values of *n* fitting to the measured datasets by the MMSE-based optimization method for the CI model are given in Table 4.

#### 3.1.2. FI Model

The FI path loss model is given by

(9) $${\mathrm{PL}}^{\mathrm{FI}}\left(l\right)\left[\mathrm{dB}\right]=\alpha +10\;\cdot \;\beta \log_{10}\left(l\right)+X_{\sigma }^{\mathrm{FI}}$$

where α is the floating-intercept in dB and this parameter is equivalent to free space path loss, and β is the slope of the line, which is similar to the PLE, $X_{\sigma }^{\mathrm{FI}}\left(\mu ,\sigma^{FI}\right)$ is the Gaussian random variable with zero mean (μ=0) and standard deviation $\sigma^{FI}$, which defines large-scale signal fluctuations about the mean path loss over the length between the transmitter and receiver. The FI method is used in the wireless world initiative new radio (WINNER) II [54] and 3rd generation partnership project (3GPP) standards [55]. Remarkably, Equation (9) expects two parameters, whereas the CI method only needs a single parameter, PLE parameter *n*. A comparative analysis between the CI and FI path loss methods causes extremely comparable shadow deteriorating default variations in mmWave outdoor channels [7,53,56,57]. The FI path loss model Equation (9) uses α as the floating intercept in dB (in contrast to an FSPL reference), and β is the slope of the line (in contrast to a PLE). Assuming $G={\mathrm{PL}}^{\mathrm{FI}}\left(d\right)\left[\mathrm{dB}\right]$, and $L=10\log_{10}\left(d\right)$, we can proceed to determine the optimized lowest level SF as

(10) $$X_{\sigma }^{\mathrm{FI}}=G-\alpha -\beta L$$

and the SF standard deviation is:

(11) $$\begin{array}{c} \sigma^{\mathrm{FI}}=\sqrt{\sum X_{\sigma }^{{\mathrm{FI}}^{2}}/D}=\sqrt{\sum {\left(G-\alpha -\beta L\right)}^{2}/D} \end{array}$$

As the smallest variation is expected for the term $\sigma^{\mathrm{FI}}$, this means the expression $\sum {\left(G-\alpha -\beta L\right)}^{2}$ is to be minimized, which means its partial derivatives with respect to α and β should be zero:

(12) $$\begin{array}{c} \partial \sum {\left(G-\alpha -\beta L\right)}^{2}\;/\;\partial \alpha =\sum 2\left(\alpha +\beta L-G\right)=2\left(D\alpha +\beta \sum L-\sum G\right)=0 \end{array}$$

(13) $$\begin{array}{c} \partial \sum {\left(G-\alpha -\beta L\right)}^{2}\;/\;\partial \beta =\sum 2L\left(\alpha +\beta L-G\right)=2\left(\alpha \sum L+\beta \sum L^{2}-\sum LG\right)=0 \end{array}$$

Equations (12) and (13) yield:

(14) $$\begin{array}{cc} \; & D\alpha +\beta \sum L-\sum G=0 \end{array}$$

(15) $$\begin{array}{cc} \; & \alpha \sum L+\beta \sum L^{2}-\sum LG=0 \end{array}$$

Combining (14) and (15), we obtain:

(16) $$\alpha =\frac{\sum L\sum LG-\sum L^{2}\sum G}{{\left(\sum L\right)}^{2}-D\sum L^{2}}$$

(17) $$\beta =\frac{\sum L\sum G-D\sum LG}{{\left(\sum L\right)}^{2}-D\sum L^{2}}$$

The optimum standard deviation of SF can be achieved by replacing α and β in (11) with (16) and (17), respectively. The mean values of all the vector elements are directly determined in the dB scale. The calculated values of α and β for the FI model are given in Table 4.

### 3.2. Multi-Frequency Propagation

A multi-frequency method can be regarded as sufficient since interior spaces exhibit frequency-dependent losses beyond the first meter due to the surrounding environment [5]. This section gives a multi-frequency model called the ”alpha-beta-gamma” model to analyze the experimentally measured attenuation datasets.

#### 3.2.1. CIF Model

In [5], it was considered that a multi-frequency method could be regarded as sufficient in the closed indoor environment as there exists frequency-dependent loss after a 1 m distance from the transmitter due to the surrounding environment [5]. The CI model can be customized to implement the frequency-dependent path loss exponent (CIF) that utilizes the same physically driven free space path loss anchor at 1m as the CI model. The path loss of the CIF method is given by

(18) $$\begin{array}{c} PL^{CIF}\left(f,d\right)\left[\mathrm{dB}\right]=L\left(f,1m\right)+\left(n\left(1-n\right)+nbf\;/\;f_{0}\right)10\;\cdot \;\log\left(d\;/\;1\;m\right)+S_{\mu ,\sigma }^{\mathrm{CIF}} \end{array}$$

where d(m) is the distance between Tx and Rx greater than 1 m, *n* is the path loss exponent (PLE) that describes the dependence of propagation loss in the path (in dB) to the logarithm of the distance starting at 1 m, and $S_{\mu ,\sigma }^{\mathrm{CIF}}$ is the Gaussian random variable with a zero mean and standard deviation σ(dB).

This random variable characterizes the large-scale channel fluctuations due to shadowing, and *b* is the optimization parameter that presents the path loss slope of the linear frequency dependence. *L*(1 m) is the free-space loss at a distance of 1 m, with $f_{c}$ being the center frequency $L_{0}\left(\mathrm{dB}\right)=20\log\left(4\pi d_{0}\;/\;\lambda \right)=32.4+20\log f_{c}\left(\mathrm{GHz}\right)$. f(GHz) is the operating carrier frequency and $f_{0}$ is the minimum investigated frequency of operating frequencies [58]. The frequency $f_{0}$ is computed as $f_{0}=\sum_{k=1}^{K}f_{k}N_{k}\;/\;\sum_{k=1}^{K}N_{k}$ where $f_{0}$ is the weighted frequency average of all measurements for each particular scene which is determined by adding all over the frequencies, the total number of recorded data $N_{k}$ at a specific frequency and antenna scenario, multiplied by the corresponding frequency $f_{k}$, and dividing that sum by the total number of measurements $\sum_{k=1}^{K}N_{k}$ taken over all frequencies for that specific scenario and the used transmitter and receiver system.

##### CIF Method: MMSE-Based Parameters

After changing the side of Equation (18), if we assume $F=PL^{CIF}\left(f,d\right)\left[\mathrm{dB}\right]-L\left(f,d_{0}\right)$, $Z=10\log(d\;/\;d_{0})$, p=n(1−b), and $q=nb\;/\;f_{0}$, we obtain:

(19) $$S_{\mu ,\sigma }^{\mathrm{CIF}}=F-Z\left(p+qf\right)$$

The SF standard deviation is:

(20) $$\sigma^{\mathrm{CIF}}=\sqrt{\sum (S_{\mu ,\sigma }^{\mathrm{CIF}}{)}^{2}/N}=\sqrt{\sum {\left(F-Z\left(p+qf\right)\right)}^{2}/N}$$

Minimizing $\sigma^{\mathrm{CIF}}$ is equivalent to minimizing $\sum {\left(F-Z\left(p+qf\right)\right)}^{2}$. When $\sum {\left(F-Z\left(p+qf\right)\right)}^{2}$ is minimized, its derivatives with respect to *p* and *q* should be zero, meaning that:

(21) $$\begin{array}{c} \partial \sum {\left(F-Z\left(p+qf\right)\right)}^{2}\;/\;\partial p=\sum 2Z\left(pZ+qZf-F\right)=2\left(p\sum Z^{2}+q\sum Z^{2}f-\sum ZF\right)=0 \end{array}$$

(22) $$\begin{array}{c} \partial \sum {\left(F-Z\left(p+qf\right)\right)}^{2}\;/\;\partial q=\sum 2Zf\left(pZ+qZf-F\right)=2\left(p\sum Z^{2}f+q\sum Z^{2}f^{2}\right.\left.-\sum ZFf\right)=0 \end{array}$$

After simplification and combination, we obtain:

(23) $$\begin{array}{cc} p & =\sum Z^{2}f\sum ZFf-\sum Z^{2}f^{2}\sum ZF\;/\;{\left(\sum Z^{2}f\right)}^{2}-\sum Z^{2}\sum Z^{2}f^{2} \end{array}$$

(24) $$\begin{array}{cc} q & =\sum Z^{2}f\sum ZF-\sum Z^{2}\sum ZFf\;/\;{\left(\sum Z^{2}f\right)}^{2}-\sum Z^{2}\sum Z^{2}f^{2} \end{array}$$

In Equations (23) and (24), the closed-loop solution of the assumed terms *p* and *q* was derived. The standard derivation of the shadow factor can be derived by inserting *p* and *q* in Equation (20). By using the initial definition p=n(1−b) and $q=nb\;/\;f_{0}$, the values of *n* and *b* can be calculated.

##### CIF Method: MMSE-Based Parameters

The calculated values of the CIF model are given in Table 4.

#### 3.2.2. ABG Model

A three-parameter multifrequency-type model known as the ABG model has terms depending on the frequency and distance to describe the propagation loss at different frequencies [53,58]. The ABG model equation is given by (25)

(25) $$\begin{array}{c} {\mathrm{PL}}^{\mathrm{ABG}}\left(f,d\right)\left[\mathrm{dB}\right]=10\alpha \log_{10}\left(\frac{d}{d_{0}}\right)+\beta +10\gamma \log_{10}\left(\frac{f}{1\mathrm{GHz}}\right)+X_{\sigma }^{\mathrm{ABG}} \end{array}$$

where α and γ are related to the path length and frequency component of the path loss of the link, *f* is the frequency in GHz, and β is a parameter used as an offset tool that lacks any physical importance. The parameter $X_{\sigma }^{\mathrm{ABG}}\left(\mu ,\sigma^{ABG}\right)$ is a Gaussian random variable describing large-scale received signal variations of the mean path loss over the path between transmitter and receiver. The ABG model might be seen as a multifrequency expansion of the FI model. It can be shown that if γ= 0 or 2 and deploying for a single frequency, it turns into an FI model. The optimum values of the α, β, and γ coefficients can be determined using the MMSE-based optimization technique. The ABG model can be similar in shape to the CI model if it can be reduced to the CI if α is equal to 20log(4π/c),β to the PLEn and γ to 2. The ABG model modifies the FI for several frequencies; however, the FI may also be achieved with a single frequency in the ABG model [28]. Since the ABG model needs three parameters, the CI model only needs one parameter, making the CI model more efficient considering the computational complexity. There are critics that claim that the additional two coefficients in the ABG model offer only minor enhancement to the overall accuracy [7,59].

The parameters’ values obtained through MMSE-based optimization can be determined by assuming $A={\mathrm{PL}}^{\mathrm{ABG}}\left(f,d\right)\left[\mathrm{dB}\right],\;L=10\log_{10}\left(d\right)$, and $R=10\log_{10}\left(f\right)$ in (25), whilst the SF is given by

(26) $$X_{\sigma }^{\mathrm{ABG}}=A-\alpha L-\beta -\gamma R$$

and the SF standard deviation is:

(27) $$\begin{array}{c} \sigma^{\mathrm{ABG}}=\sqrt{\sum X_{\sigma }^{{\mathrm{ABG}}^{2}}/D}=\sqrt{\sum {\left(A-\alpha L-\beta -\gamma R\right)}^{2}/D} \end{array}$$

As the smallest variation is expected for the term $\sigma^{\mathrm{ABG}}$, which means that the expression $\sum {\left(A-\alpha L-\beta -\gamma R\right)}^{2}$ is to be zero, which can be realized through the partial derivatives of α,β, and setting γ to zero:

(28) $$\begin{array}{c} \partial \sum {\left(A-\alpha L-\beta -\gamma R\right)}^{2}\;/\;\partial \alpha =\sum 2L\left(\alpha D+\beta +\gamma R-A\right)=2\left(\alpha \sum L^{2}+\beta \sum L\right.\left.+\gamma \sum LR-\sum LA\right)=0 \end{array}$$

(29) $$\begin{array}{c} \partial \sum {\left(A-\alpha L-\beta -\gamma R\right)}^{2}\;/\;\partial \beta =\sum 2\left(\alpha L+\beta +\gamma R-A\right)=2\left(\alpha \sum L+D\beta \right.\left.\gamma \sum R\right.\left.-\sum A\right)=0 \end{array}$$

(30) $$\begin{array}{c} \begin{array}{c} \partial \sum {\left(A-\alpha L-\beta -\gamma R\right)}^{2}\;/\;\partial \gamma =\sum 2R\left(\alpha L+\beta +\gamma R-A\right)=2\left(\alpha \sum LR+\beta \sum R\right.+\left.\gamma \sum R^{2}-\sum RA\right)=0 \end{array} \end{array}$$

from (28)–(30), it is clear that:

(31) $$\begin{array}{c} \alpha \sum L^{2}+\beta \sum L+\gamma \sum LR-\sum LA=0 \end{array}$$

(32) $$\begin{array}{c} \alpha \sum L+D\beta +\gamma \sum R-\sum A=0 \end{array}$$

(33) $$\begin{array}{c} \alpha \sum LR+\beta \sum R+\gamma \sum R^{2}-\sum R=0 \end{array}$$

The numeric values of α, β, and γ for the ABG model can be calculated by solving the matrix (34):

(34) $$\left(\begin{array}{c} \alpha \\ \beta \\ \gamma \end{array}\right)={\left(\begin{array}{ccc} \sum L^{2} & \sum L & \sum LR\\ \sum L & D & \sum R\\ \sum LR & \sum R & \sum R^{2} \end{array}\right)}^{-1}\left(\begin{array}{c} \sum LA\\ \sum A\\ \sum RA \end{array}\right)$$

The calculated coefficients of the ABG model are given in Table 4.

## 4. Analysis of the Large-Scale Path Loss Models

Measurement campaigns at 3.7 and 28 GHz were carried out to examine the university campus’s long-term path loss models’ coefficients. Under LOS conditions, the received signal strength was measured with sophisticated devices, as explained earlier. The following long-term path loss models: CI, FI, and ABG were used in this work. At two frequencies, 3.7 and 28 GHz, three different environments were examined. The measurement drive was carried out in a 100-m-long hallway, in the first case in H–H co-polarization and the second on the same floor, but the receiver was totally inside the closed computer lab. Table 4 and Table 5 show the coefficients of the FI, CI, CIF, and ABG models and the standard deviation (σ) values, respectively.

**Figure 6:**
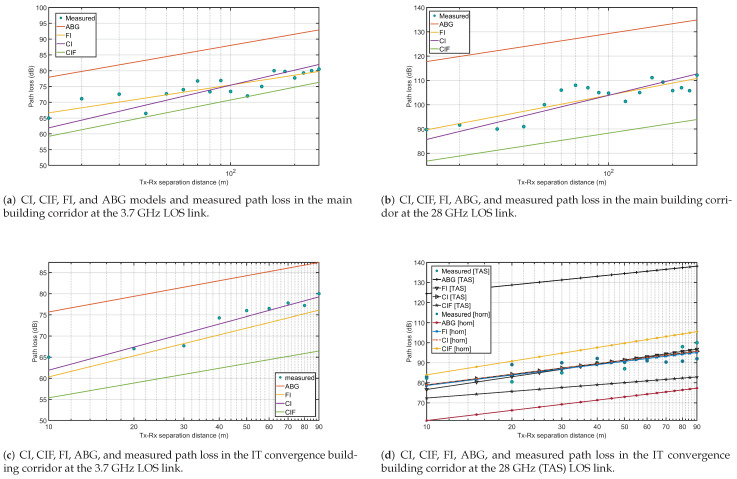
The figure depicts CI, CIF, FI, ABG, and the measured path loss in LOS link at the frequency of 3.7 GHz (**a**,**c**) and at 28 GHz (**b**,**d**).

**Figure 7:**
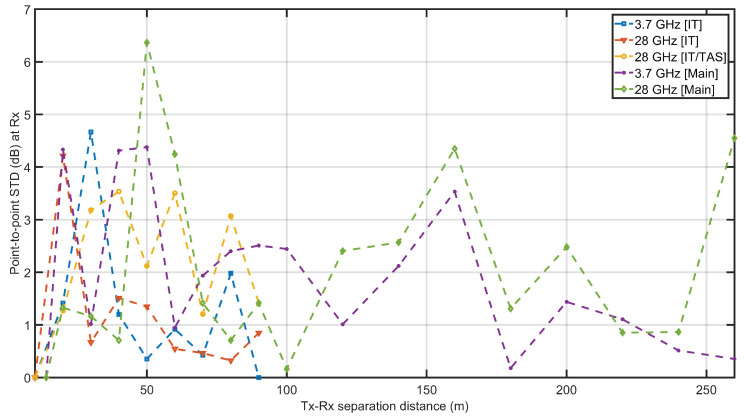
The figure depicts the point-to-point standard deviation of recorded data at 3.7 and 28 GHz under various antenna settings.

Figure 6a–d depict a logarithmic scale study of measured path loss in the hallway through the CI, FI, and ABG models at the 3.7 and 28 GHz frequencies. A comparison of the path loss-derived CI, CIF, FI, and ABG models with the observed results was computed, along with the standard deviation. In Figure 6a, the measured and predicted attenuation by the FI, CI, CIF, and ABG models was plotted for the measured data at the main building corridor at 3.7 GHz. The figure show that the CI and FI model fits to the path loss attenuation are close to the measured results whereas the attenuation predicted by the CIF and ABG were not well fitted to the measured data. In Figure 6b, the measured and the predicted attenuation by the FI, CI, CIF, and ABG models was plotted for the measured data at the main building corridor at 28 GHz. The figure show that the CI and FI model fits to the path loss attenuation are close to the measured results, whereas the attenuation predicted by the CIF and ABG were not well fitted to the measured data. In Figure 6c, the measured and the predicted attenuation by the FI, CI, CIF, and ABG models was plotted for the measured data at the IT convergence building corridor at 3.7 GHz. The figure shows that the CI and FI model fits to the path loss attenuation are close to the measured results, whereas the attenuation predicted by the CIF and ABG were not well fitted to the measured data. In Figure 6d, the measured and the predicted attenuation by the FI, CI, CIF, and ABG models was plotted for the measured data at the IT convergence building corridor at 28 GHz using horn–horn and horn–TAS antenna system, respectively, at the transmitter and receiver ends. The figure shows that the CI and FI model fits to the path loss attenuation for both the horn–horn and horn–TAS antenna combinations are close to the measured results, whereas the attenuation predicted by the CIF and ABG were not well fitted to the measured data. The standard deviation of the CI model was 3.046, 4.057 in the main building corridor, and 1.616, 2.936, 4.638, respectively, for the frequencies of 3.7 GHz (horn), 28 GHz (horn), and 28 GHz (TAS). The standard deviation of the CIF model was 9.100, 6.934 in the main building corridor, and 11.514, 3.312, respectively, for the frequencies of 3.7 GHz (horn), 28 GHz (horn), and 28 GHz (TAS). For the FI model, the standard deviation was 2.257, 3.679 in the main building corridor, and 1.541, 2.896 in the IT convergence building corridor, respectively, for the frequencies of 3.7 GHz (horn), 28 GHz (horn), and 28 GHz (TAS). For the ABG model, the standard deviation was 14.238, 14.531 in the main building corridor, and 9.967, 6.893 in the IT convergence building corridor, respectively, for the frequencies of 3.7 GHz (horn), 28 GHz (horn), and 28 GHz (TAS). Figure 7 presents the point-to-point (P2P) standard deviations of the collected data in H–H polarization for several experiments at frequencies of 3.7 and 28 GHz. In the IT building corridor, the P2P fluctuation is 4.5, 4.2, 3.4 dB, respectively, at 3.7, 28, and 28 GHz (TAS) frequencies. However, in the main building corridor, the P2P fluctuation is 4.2, 6.4 dB, respectively, at frequencies of 3.7, 28 GHz.

## 5. Results and Discussions

The measured data pattern for the two extended corridors demonstrate that the environment impacts the path loss. In both of the corridors, the single-frequency model CI and FI showed almost identical performance while the multi-frequency models CIF and ABG did not show satisfactory performance compared to the measured path loss. Furthermore, if we consider the distribution of the shadowing factor of the single frequency to the multi-frequency, the multi-frequency shadowing factors are more widely compared to the single-frequency models.

## 6. Conclusions

This study focused on the comparative performance of large-scale channel models that characterize wireless path loss in long indoor corridors that have not been discussed in the literature. According to the outcome of this study, the CI and FI path loss models showed almost identical behavior in two long indoor corridors for LOS horn antenna links. This shadowing factor and point-by-point standard deviations were investigated to circumscribe the signal variability per 10 m distance from the transmitter to the receiver. The standard deviation of the path loss parameters at the main building corridor (370 m) were higher than the IT convergence building corridor (90 m) at the frequency 3.7 GHz. However, at the 28 GHz frequency, the difference in the standard deviation of the path loss can be ignored. The propagation of radio waves in the hallway is intense due to the proximity of the walls and the type of material utilized in the walls, floor material, roof, and panels. The shadow fading achieved by our obtained results is higher than the values suggested by the other experiments.

## Figures and Tables

**Table 1:** Indoor corridor propagation study information for the path length in the corridor greater than 30 m.

Ref.	Link	Frequency (GHz)	Distance (m)
[32]	N/LOS ${}^{∔}$	60	30
[26]	N/LOS	14/22	30
[34]	LOS	39	5/50
[35]	LOS	28	1/60
[36]	N/LOS	60	2.4/60
[27]	N/LOS	26/32/39	65
[37,38,39]	N/LOS	28/38	1/67
[40]	N/LOS	41/0.5	1.35/70
[41,42]	N/LOS	60/74	10/80
[41]	N/LOS	30	10/80
[33]	N/LOS	28	<100
This work	N/LOS	3.7/28	90/260

${}^{∔}$ N/LOS is used to mean both non-line-of-sight and line-of-sight links.

**Table 2:** Parameter specifications of the channel.

Parameters	3 GHz	28 GHz	28 GHz ${}^{†}$
Operating frequency (GHz)	3.7	28	28
Bandwidth (MHz)	1	1	1
Tx antenna	horn	horn	horn
Rx antenna	${}^{‡}$	${}^{‡‡}$	${}^{⋊}$
LNA gain (dB)	57	57	57
System gain (dB)	40	40	40
Tx antenna height (m)	1.75	1.75	1.75
Rx antenna height (m)	1.5	1.5	1.5
Tx antenna gain	10	20	20
Rx antenna gain	10	20	20
Beamwidth	45–45${}^{\circ }$	18–21${}^{\circ }$	18–21${}^{\circ }$
Polarization	H	H	H
Tx cable loss (dB)	2.8	9.4	9.4
Rx cable loss (dB)	2	6.2	6.2

${}^{†}$ Tracking antenna system at the Rx end; ${}^{‡}$ double-ridged wave-guide horn antenna (typical gain: 10 dBi); ${}^{‡‡}$ 20 dBi WR28 standard wave-guide horn antenna; ${}^{⋊}$ 20 dBi WR28 standard wave-guide horn antenna 16×2 array system.

**Table 3:** Materials used in the main and IT convergence building.

Location	Items	Materials
Main	Floor	Concrete tiles
	Wall	Concrete + cement
	Ceiling	Styrofoam supported by suspended
	Door	Metal
	Window	Glass
	Height × width × length	3.43 m × 2.9 m × 375 m
IT	Floor	Concrete tiles
	Ceiling	Styrofoam supported by suspended
	Wall	Cement + concrete
	Door	Metal
	Window	Glass (structure metal)
	Height × width × length	2.7 m × 3.547 m × 90 m

**Table 4:** Parameters of different propagation techniques.

Locat.	Freq. ${}^{◃}$	CI	FI (α)	CIF	ABG (β)	CI (*n*)	FI (β)	CIF (*n*)	ABG (α)	CIF (*b*)	ABG (γ)
IT	3.7	43.810	46.522	43.810	29.507	1.830	1.668	1.692	1.439	0.028	5.563
	28	61.380	58.691	61.380	29.507	1.763	1.924	1.692	1.439	0.028	5.563
	28 ${}^{▹}$	61.380	55.578	61.380	29.507	1.760	2.108	1.692	1.439	0.028	5.563
Main	3.7	43.810	54.815	43.810	37.117	1.582	1.033	1.773	1.349	0.051	4.504
	28	61.380	70.593	61.380	37.117	2.125	1.666	1.773	1.349	0.051	4.504

${}^{◃}$ frequency is in GHz; ${}^{▹}$ tracking antenna system.

**Table 5:** Shadow factor of the CI, CIF, FI, and ABG models obtained through the MMSE technique.

Locat.	Freq.	CI (σ)	FI (σ)	CIF (σ)	ABG(σ)
IT	3.7 GHz	1.616	1.541	11.514	9.967
	28 GHz	2.936	2.896	3.312	6.893
	28 GHz (TAS)	4.638	4.520	4.712	7.459
Main	3.7 GHz	3.046	2.257	9.100	14.238
	28 GHz	4.057	3.679	6.934	14.531

## Data Availability

Not applicable.
